# Case report: Post-thoracoscopy pendelluft monitoring

**DOI:** 10.3389/fmed.2022.1108637

**Published:** 2023-03-02

**Authors:** Zhibin Xiao, Na Zhang, Xiajing Zhang, Wenjun Lu, Changjun Gao, Xude Sun

**Affiliations:** ^1^Department of Anesthesiology, The Second Affiliated Hospital of Air Force Medical University, Xi'an, Shaanxi, China; ^2^Department of Thoracic Surgery, The Second Affiliated Hospital of Air Force Medical University, Xi'an, Shaanxi, China; ^3^Department of Anesthesiology, The 986th Air Force Hospital, Xijing Hospital, The Air Force Medical University, Xi'an, Shaanxi, China; ^4^Institute of Medical Research, Northwestern Polytechnical University, Xi'an, Shaanxi, China

**Keywords:** electrical impedance tomography, pendelluft, post-thoracoscopy, lung injury, pulmonary ventilation

## Abstract

Asynchronous alveolar ventilation is called pendelluft, which may induce lung injury in spontaneously breathing patients. We report a case that electrical impedance tomography (EIT) was used to assess the pendelluft in a post-thoracoscopy patient. The pendelluft amplitude was as high as 77.5% of the tidal variation. The average regional time shift was 0.5 s. The patient was instructed to adjust the breathing method, symptomatic treatment was performed, and the symptoms were improved. This is the first case reporting pendelluft in a post-thoracoscopy patient. Our case demonstrated that (1) pendelluft may occur in post-thoracoscopy patients and it effects lung function, and (2) early identification of affected patients and implementation of corresponding treatments could improve patient outcomes.

## Introduction

In recent years, lung protective ventilation strategies during the perioperative period have been widely used. However, post-operative pulmonary complications can occur from time to time (1, 2). Post-operative pulmonary function recovery can also be time-consuming. Additionally, individualized pulmonary rehabilitation training has not been popularized, one important reason being the current lack of effective clinical methods to assess intrapulmonary ventilation in different regions (2). Pulmonary function defect may occur after minimal invasive surgery, which was not systematically assessed (3). Once the patient recovers spontaneously breathing after surgery, non-uniform transmission of pleural pressure generated by diaphragmatic contraction may cause a phenomenon called pendelluft (4). In the extreme cases such as flail chest, pendelluft volume can be as high as 12.5% of the total volume passing through an airway (5). Although pendelluft could be potentially harmful by introducing local overstretch, tidal recruitment and inflammation, up to now, no traditional technique can track the pendelluft level.

Electrical impedance tomography (EIT) is a novel bedside imaging modality that provides continuous images of pulmonary ventilation and shows regional ventilation changes (6). EIT has been proposed to assess the pulmonary rehabilitation program and inspiratory muscle activities (7, 8), as well as capture the amplitude of pendelluft (9).

We present a case in which significant pendelluft was captured in a patient after thoracoscopy detected by EIT. Pulmonary rehabilitation was conducted for the patient who otherwise would have been discharged. Improvement in pulmonary function was observed after the early intervention.

## Case description

A 49-year-old male patient with 10 years history of diabetes was admitted to our hospital. Ground-glass opaque nodules and dense nodular shadows were found in the posterior segment of the upper right lobe of the lung during computed tomography examination. A single-port thoracoscopy resection of S2b + lymph node sampling of the right upper lobe was performed under general anesthesia. The surgery lasted 1 h 55 min and was conducted successfully.

**Figure 1:**
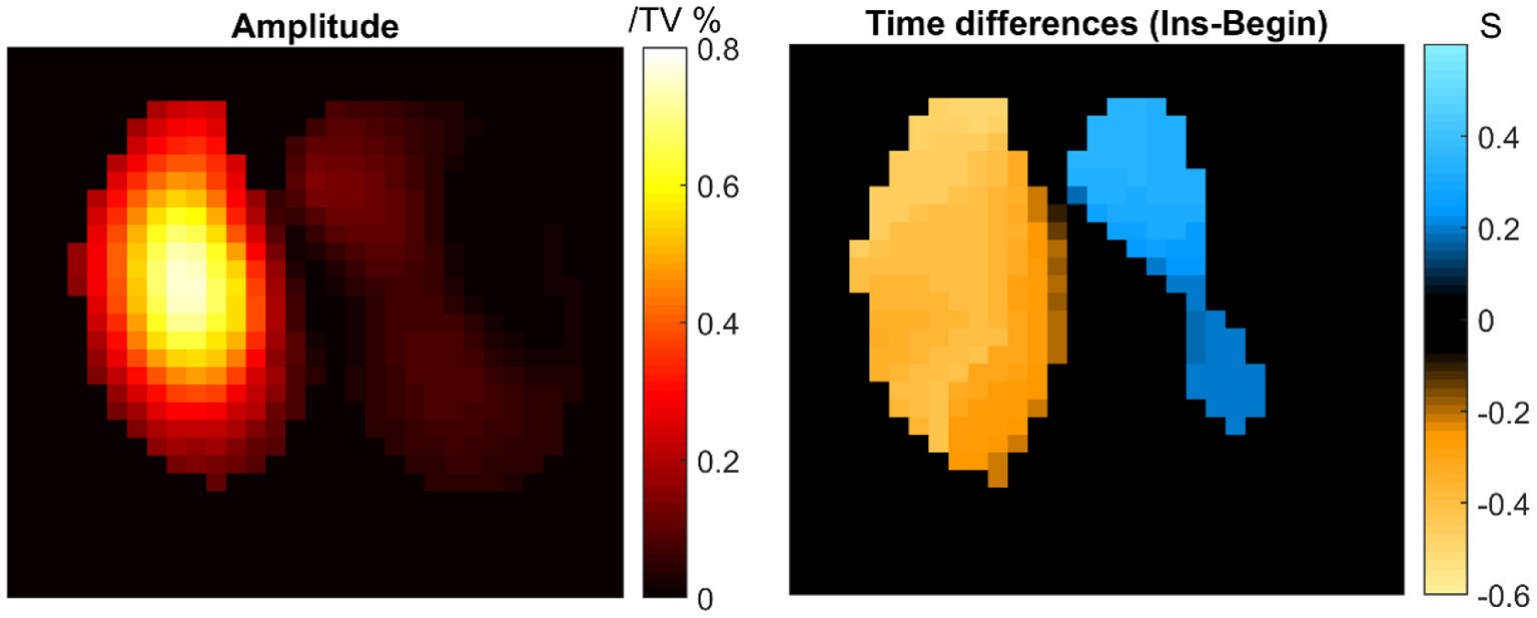
Pendelluft amplitude and time differences calculated according to a previous study (9). TV, tidal variation.

During the follow-up on the day after surgery, the patient had rapid, shallow breathing and was irritable. EIT measurement was conducted (VenTom-100, MidasMED Biomedical technology, Suzhou, China). A belt with 16 equidistantly fixed electrodes was placed around the chest in one transverse plane at the level of the 4th intercostal space at the parasternal line. Raw EIT data were acquired with at a scan rate of 20 images/s using excitation currents of 1 mArms applied through opposite electrodes. Image reconstruction was accomplished by the GREIT algorithm (10). In brief, inspiration and expiration were identified for global and regional impedance-time curves. The impedance differences between the sum of all regional tidal impedance variation (TIV) and the global TIV represented the pendelluft volume. The time shift between the regional and global inspiration start denoted as the pendelluft-induced time differences. EIT revealed asynchronous breathing in the right and left lungs with obvious pendelluft breathing pattern (Figure 1). The pendelluft amplitude was as high as 77.5% of the tidal variation. The average regional time shift was 0.5 s. We suspected that the airways were partially blocked by the sputum, which significantly increased the airway resistance heterogeneously.

To further clarify the patient's lung status, he was then scheduled for a computed tomography examination, the results of which suggested: subcutaneous emphysema in the right thoracolumbar region, right hydropneumothorax and a small amount of fluid in the left pleural cavity, and a patchy high-density shadow in the surgical area of the right lung. The patient was advised to cough up more sputum and practice deep breathing exercises more frequently. After three days of anti-infection, expectorant, and analgesic symptomatic treatment, the patient had a respiratory rate of 24 bpm with no symptoms of chest tightness or dyspnoea, though he still felt shortness of breath after physical activities. The patient was discharged after the removal of the chest drainage tube.

**Figure 2:**
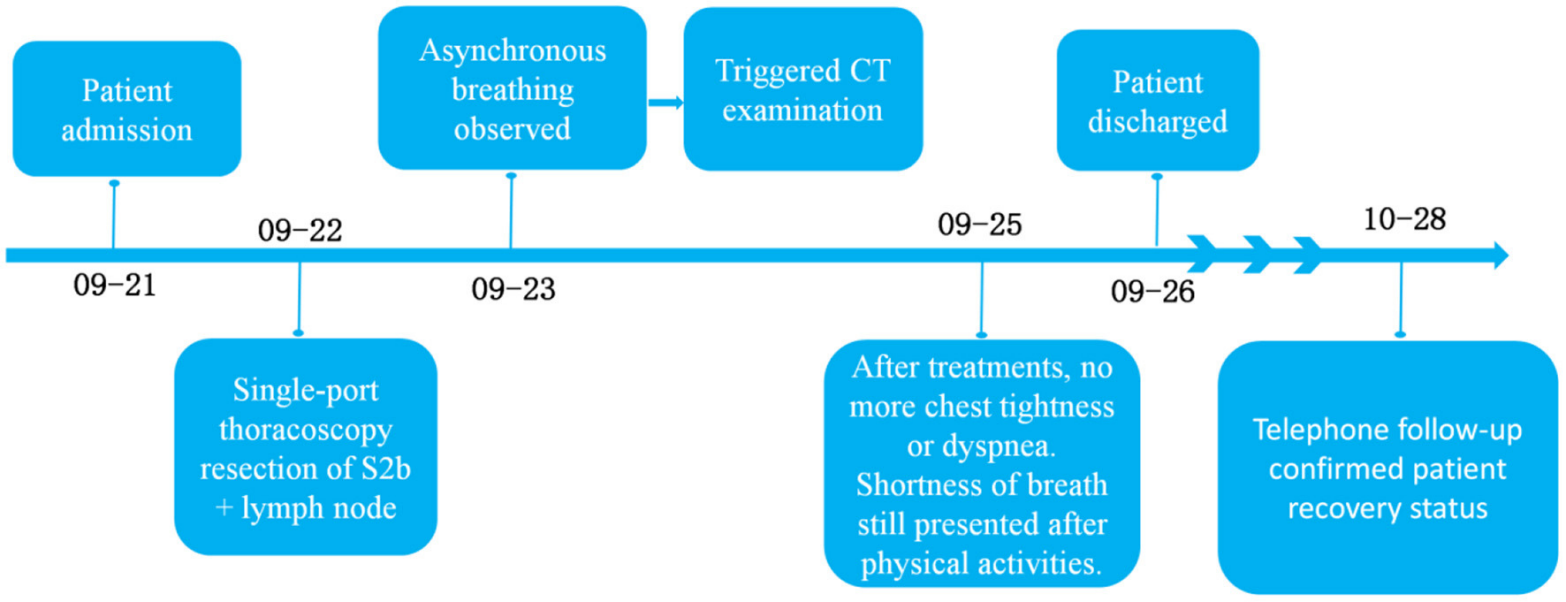
Timeline of patient treatment.

**Timeline:** Figure 2 showed information about the treatment process.

## Diagnostic assessment

Telephone follow-up one month after the operation: the patient felt good, but he had shortness of breath after fast walking. Unfortunately, due to the COVID-19 epidemic, it was inconvenient for the patient to come for re-examination.

## Discussion

The pendelluft is a phenomenon of gas redistribution in the lungs due to asynchronous alveolar ventilation. It was found that for patients with acute respiratory failure or intrapulmonary injury who are mechanically ventilated in the ICU, preserving moderate spontaneous breathing can help improve ventilation in the gravity-dependent zone, increase oxygenation, and prevent diaphragmatic atrophy. However, excessive inspiratory efforts have the potential to exacerbate lung injury through the pendelluft mechanism (11, 12). Since the pendelluft phenomenon involves only intrapulmonary gas redistribution and does not change the tidal volume *per se*, it means that in critically ill patients with lung injury, even if a small tidal volume lung-protective ventilation strategy has been adopted, the pendelluft phenomenon may still cause local regional lung hyperinflation and become a hidden source of lung injury exacerbation (13). During thoracoscopy, one-lung ventilation with positive pressure combined with the extended resection of the S2b in the upper lobe of the right lung may result in a non-uniform transfer of pleural pressure from diaphragmatic contraction. Such non-uniform pleural pressure may cause pendelluft. Due to the limitation of currently well-established techniques, the presence of pendelluft was not reported so far in patients after thoracoscopy (14). Follow-up treatment for the post-thoracoscopy patients could not be provided accordingly. As the first report of its kind, we took advantage of EIT as a bedside tool to detect the pendelluft in timely manner, helping with early treatment and intervention. The patient was instructed to adjust the breathing method, symptomatic treatment was performed, and the abnormal breathing phenomenon disappeared.

The EIT imaging technique has its known limitations: EIT image does not provide the precise anatomical localization of lung tissue and the spatial resolution is relatively low compared to computed tomography or magnetic resonance imaging. Electrode belt cannot be attached to the thorax for EIT measurement when the patients' wounds are around 4th intercostal space after surgery. As a limitation of the study, quantitative assessment of the treatment outcomes was not recorded. Nevertheless, our case demonstrated that (1) pendelluft may occur in post-thoracoscopy patients and it effects lung function, and (2) early identification of affected patients and implementation of corresponding treatments could improve patient outcomes. Further studies are warranted to explore the incidence of pendelluft in such patient group. Randomized controlled trial could be conducted to compare the post-operation recovery with and without EIT-based pendelluft assessment. *But EIT does have a very high temporal resolution, which makes the functional imaging possible. In fact, the calculation utilizes the high temporal resolution to estimate the pendelluft phase shift and thereby the corresponding amplitude*.

## Conclusion

EIT can be used to detect the pendelluft phenomenon in post-thoracoscopy patients at the bedside, which provides important evidence for symptomatic diagnosis and treatment.

## Data availability statement

The original contributions presented in the study are included in the article/supplementary material, further inquiries can be directed to the corresponding authors.

## Ethics statement

The studies involving human participants were reviewed and approved by Ethics Committee of Tangdu Hospital of Air Force Military Medical University. The patients/participants provided their written informed consent to participate in this study. Written informed consent was obtained from the individual(s) for the publication of any potentially identifiable images or data included in this article.

## Author contributions

XS and CG conceived and designed the study. ZX and NZ performed of the experiments and drafted the manuscript. XZ edited the manuscript. WL analyzed the data. All authors have read and approved the final version of the manuscript.
